# Ichneumonidae (Hymenoptera) species new to the fauna of Norway

**DOI:** 10.3897/BDJ.2.e1047

**Published:** 2014-02-25

**Authors:** Andrei E. Humala, Alexey Reshchikov

**Affiliations:** † University of Oslo, Natural History Museum, Department of Research and Collections, Oslo, Norway; ‡ Forest Research Institute KRC RAS, Petrozavodsk, Russia; § Swedish Museum of Natural History, Stockholm, Sweden

**Keywords:** Ichneumon wasps, Ichneumonidae, Norway, Scandinavia, new records

## Abstract

The present paper contains new distributional records for 61 species of ichneumon wasps (Hymenoptera, Ichneumonidae) previously unknown for Norway, six of them are reported from Scandinavia for the first time.

## Introduction

The Ichneumonidae are the largest family within Hymenoptera, and one of the largest among insects. Due to the great diversity and difficulties in identification of many species in this group, our knowledge of the Norwegian fauna remains insufficient. During the last twenty years, more than 500 species of Ichneumonidae were reported as new to the fauna of Norway (Kopelke 1994, Riedel and Berg 1997, Riedel et al. 2000, Horstmann 2004, Riedel et al. 2005, Riedel and Hansen 2007, Kasparyan and Kopelke 2009, Hansen et al. 2010, Kasparyan and Kopelke 2010, Humala and Reshchikov 2012, Riedel and Hansen 2012, Reshchikov 2013, Riedel and Hansen 2013). Although in total more than 1700 species of Ichneumonidae have been listed for Norway so far, the published checklists from adjacent territories (Koponen et al. 1995, Broad 2011) suggest that the Norwegian list is still very incomplete.

## Materials and methods

**Figure 1.**
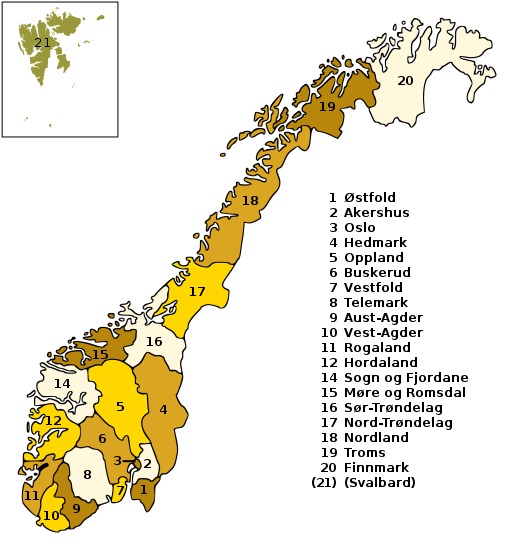
The division of Norway in administrative regions (counties).

During the 2011 field season, ichneumonids were sampled in many localities across the southern half of the country. The division of Norway in administrative regions (counties) applied in faunistic studies is shown on the map (Fig. 1). The northern-most provinces of Norway were not sampled due to time constraints of the project. The light-weight Malaise traps were the main method of collecting, although yellow pan traps and netting were also used.

The original collected material and voucher specimens for DNA barcoding are deposited at the Natural History Museum, University of Oslo, Norway.

Additional material was borrowed from the following museums: The Museum of Natural History and Archaeology, Trondheim; University Museum of Bergen; reared material of the genus *Saotis* Förster, 1869 was provided by Department of Biology, University of Eastern Finland. Species identification in subfamily Ctenopelmatinae was confirmed by comparison with the type specimens deposited in Swedish museums (The Swedish Museum of Natural History, Stockholm; Zoological Institute of Uppsala University; Zoological Institute, Lund). All taxa are listed in alphabetic order according to the recent Catalogue of World Ichneumonidae (Yu et al. 2012). Species distributions are based on the same catalogue and Fauna Europaea website (Achterberg van and Zwakhals 2004) with minor changes. The terms Palaearctic and Western Palaearctic are used to specify how wide is distribution. Species distributions in Fennoscandia, in the countries adjacent or faunistically similar to Norway are given in more detail after biogeographic region name. The following abbreviations are used in the text:

NTNU – Trondheim Museum of Natural History and Archaeology (Vitenskapsmuseet)

NW – North-Western

UEF – University of Eastern Finland

ZMUN – Natural History Museum, University of Oslo

## Species new to Norway

### Acaenitinae

Förster, 1869

### Coleocentrini

Clément, 1938

### Coleocentrus caligatus

Gravenhorst, 1829

#### Materials

- **Type status:** Other material. **Occurrence:** recordedBy: Thorstensen; individualCount: 1; sex: female; **Taxon:** order: Hymenoptera; family: Ichneumonidae; genus: Coleocentrus; specificEpithet: caligatus; scientificNameAuthorship: Gravenhorst, 1829; **Location:** country: Norway; stateProvince: Aust-Agder; verbatimLocality: Risør; **Identification:** identifiedBy: Andrei E. Humala; **Event:** eventDate: 28.VI.1906; **Record Level:** collectionCode: ZMUN

#### Distribution

Palaearctic; Finland, Sweden and NW Russia (Karelia).

### Adelognathinae

Thomson, 1888

### Adelognathus difformis

Holmgren, 1857

#### Materials

- **Type status:** Other material. **Occurrence:** recordedBy: L.O. Hansen; individualCount: 1; sex: female; **Taxon:** order: Hymenoptera; family: Ichneumonidae; genus: Adelognathus; specificEpithet: difformis; scientificNameAuthorship: Holmgren, 1857; **Location:** country: Norway; stateProvince: Akerhus; verbatimLocality: Ullensaker, Sessvollmoen, Aurtjernet W; verbatimElevation: 200 m; **Identification:** identifiedBy: Andrei E. Humala; **Event:** samplingProtocol: Malaise trap; eventDate: 30.VIII-1.X.2008; habitat: sandy pine forest; **Record Level:** institutionCode: ZMUN

#### Distribution

Holarctic; Sweden, Finland and NW Russia (Leningrad region).

### Adelognathus rufithorax

Kasparyan, 1990

#### Materials

- **Type status:** Other material. **Occurrence:** recordedBy: Andrei E. Humala & Alexey Reshchikov; individualCount: 1; sex: female; **Taxon:** order: Hymenoptera; family: Ichneumonidae; genus: Adelognathus; specificEpithet: rufithorax; scientificNameAuthorship: Kasparyan, 1990; **Location:** country: Norway; stateProvince: Sogn og Fjordane; verbatimLocality: Leikanger, Kvinnefossen waterfall; verbatimElevation: 60 m; verbatimLatitude: 61°12'30.2" N; verbatimLongitude: 06°38'52.0" E; **Identification:** identifiedBy: Andrei E. Humala; **Event:** samplingProtocol: Malaise trap; eventDate: 6.VII-30.VIII.2011; habitat: spruce forest with juniper; **Record Level:** institutionCode: ZMUN

#### Distribution

Palaearctic; NW Russia (Leningrad region).

#### Notes

New to Scandinavia.

### Adelognathus stelfoxi

Fitton, Gauld & Shaw, 1982

#### Materials

- **Type status:** Other material. **Occurrence:** recordedBy: L.O. Hansen; individualCount: 1; sex: female; **Taxon:** order: Hymenoptera; family: Ichneumonidae; genus: Adelognathus; specificEpithet: stelfoxi; scientificNameAuthorship: Fitton, Gauld & Shaw, 1982; **Location:** country: Norway; stateProvince: Akerhus; verbatimLocality: Ullensaker, Sessvollmoen W; verbatimElevation: 204 m; **Identification:** identifiedBy: Andrei E. Humala; **Event:** samplingProtocol: Malaise trap; eventDate: 26.VI-25.VIII.2007; habitat: sandy pine forest; **Record Level:** institutionCode: ZMUN

#### Distribution

Palaearctic; Finland.

### Banchinae

Wesmael, 1845

### Glyptini

Cushman & Rohwer, 1920

### Glypta (Glypta) consimilis

Holmgren, 1860

#### Materials

- **Type status:** Other material. **Occurrence:** recordedBy: Andrei E.Humala & Alexey Reshchikov; individualCount: 1; sex: female; **Taxon:** order: Hymenoptera; family: Ichneumonidae; genus: Glypta; subgenus: Glypta; specificEpithet: consimilis; scientificNameAuthorship: Holmgren, 1860; **Location:** country: Norway; stateProvince: Aust-Agder; verbatimLocality: Lillesand, Froholt; verbatimElevation: 53 m; verbatimLatitude: 58°19'33.9" N; verbatimLongitude: 08°18'15.5" E; **Identification:** identifiedBy: Andrei E. Humala; **Event:** samplingProtocol: Malaise trap; eventDate: 30.VI-23.VIII.2011; habitat: oak forest; **Record Level:** institutionCode: ZMUN

#### Distribution

Holarctic; Finland, Sweden, Lithuania and NW Russia (Leningrad region).

### Campopleginae

Förster, 1869

### Leptocampoplex cremastoides

(Holmgren, 1860)

#### Materials

- **Type status:** Other material. **Occurrence:** recordedBy: Andrei E. Humala & Alexey Reshchikov; individualCount: 1; sex: female; **Taxon:** order: Hymenoptera; family: Ichneumonidae; genus: Leptocampoplex; specificEpithet: cremastoides; scientificNameAuthorship: (Holmgren, 1860); **Location:** country: Norway; stateProvince: Aust-Agder; verbatimLocality: Lillesand, Froholt; verbatimElevation: 53 m; verbatimLatitude: 58°19'33.9" N; verbatimLongitude: 08°18'15.5" E; **Identification:** identifiedBy: Andrei E. Humala; **Event:** samplingProtocol: Malaise trap; eventDate: 30.VI-23.VIII.2011; habitat: oak forest; **Record Level:** institutionCode: ZMUN
- **Type status:** Other material. **Occurrence:** recordedBy: Andrei E. Humala & Alexey Reshchikov; individualCount: 1; sex: female; associatedSequences: voucher for DNA barcoding AH057; **Taxon:** order: Hymenoptera; family: Ichneumonidae; genus: Leptocampoplex; specificEpithet: cremastoides; scientificNameAuthorship: (Holmgren, 1860); **Location:** country: Norway; stateProvince: Aust-Agder; verbatimLocality: Lillesand, Vestre Grimevann lake; verbatimLatitude: 58°19'23.1" N; verbatimLongitude: 08°20'09.1" E; **Identification:** identifiedBy: Andrei E. Humala; **Event:** samplingProtocol: Malaise trap; eventDate: 29.VI-23.VIII.2011; habitat: oak forest; **Record Level:** institutionCode: ZMUN

#### Distribution

Holarctic; Finland, Sweden and NW Russia (Karelia).

### Rhimphoctona (Xylophylax) rufocoxalis

(Clément, 1924)

#### Materials

- **Type status:** Other material. **Occurrence:** recordedBy: Kaare Aagaard; individualCount: 1; sex: female; **Taxon:** order: Hymenoptera; family: Ichneumonidae; genus: Rhimphoctona; subgenus: Xylophylax; specificEpithet: rufocoxalis; scientificNameAuthorship: (Clément, 1924); **Location:** country: Norway; stateProvince: Nord Trøndelag; verbatimLocality: Høylandet, Skiftesåa; **Identification:** identifiedBy: Andrei E. Humala; **Event:** samplingProtocol: Malaise trap; eventDate: 2.VII.1987; **Record Level:** institutionCode: NTNU
- **Type status:** Other material. **Occurrence:** recordedBy: Kaare Aagaard; individualCount: 2; sex: females; **Taxon:** order: Hymenoptera; family: Ichneumonidae; genus: Rhimphoctona; subgenus: Xylophylax; specificEpithet: rufocoxalis; scientificNameAuthorship: (Clément, 1924); **Location:** country: Norway; stateProvince: Nord Trøndelag; verbatimLocality: Høylandet, Skiftesåa; **Identification:** identifiedBy: Andrei E. Humala; **Event:** samplingProtocol: Malaise trap; eventDate: 22.VII.1987; **Record Level:** institutionCode: NTNU
- **Type status:** Other material. **Occurrence:** recordedBy: Kaare Aagaard; individualCount: 1; sex: female; **Taxon:** order: Hymenoptera; family: Ichneumonidae; genus: Rhimphoctona; subgenus: Xylophylax; specificEpithet: rufocoxalis; scientificNameAuthorship: (Clément, 1924); **Location:** country: Norway; stateProvince: Nord Trøndelag; verbatimLocality: Høylandet, Tverrråa; verbatimElevation: 340 m; **Identification:** identifiedBy: Andrei E. Humala; **Event:** samplingProtocol: Malaise trap; eventDate: 30.VII.1986; **Record Level:** institutionCode: NTNU

#### Distribution

Palaearctic; Sweden and NW Russia (Karelia).

### Cryptinae

Kirby, 1837

### Cryptini

Kirby, 1837

### Ateleute linearis

Förster, 1871

#### Materials

- **Type status:** Other material. **Occurrence:** recordedBy: Andrei E. Humala & Alexey Reshchikov; individualCount: 1; sex: male; associatedSequences: voucher for DNA barcoding AH056; **Taxon:** order: Hymenoptera; family: Ichneumonidae; genus: Ateleute; specificEpithet: linearis; scientificNameAuthorship: Förster, 1871; **Location:** country: Norway; stateProvince: Aust-Agder; verbatimLocality: Lillesand, Vestre Grimevann lake; verbatimLatitude: 58°19'23.1" N; verbatimLongitude: 08°20'09.1" E; **Identification:** identifiedBy: Andrei E. Humala; **Event:** samplingProtocol: Malaise trap; eventDate: 29.VI-23.VIII.2011; habitat: oak forest; **Record Level:** institutionCode: ZMUN

#### Distribution

Western Palaearctic; Finland, Sweden and NW Russia (Karelia, Leningrad region).

### Ctenopelmatinae

Förster, 1869

### Ctenopelmatini

Förster, 1869

### Ctenopelma boreale

Holmgren, 1857

#### Materials

- **Type status:** Other material. **Occurrence:** recordedBy: Ole Lønnve; individualCount: 1; sex: male; **Taxon:** order: Hymenoptera; family: Ichneumonidae; genus: Ctenopelma; specificEpithet: boreale; scientificNameAuthorship: Holmgren, 1857; **Location:** country: Norway; stateProvince: Hedmark; verbatimLocality: Vestmarka; **Identification:** identifiedBy: Alexey Reshchikov; **Event:** eventDate: 5.VII.1997; **Record Level:** institutionCode: ZMUN

#### Distribution

Palaearctic; Sweden, Finland and NW Russia (Karelia).

#### Notes

According to Kasparyan (Kasparyan and Khalaim 2007) *Ctenopelma boreale* Holmgren is a distinct species.

### Ctenopelma lapponicum

Holmgren, 1857

#### Materials

- **Type status:** Other material. **Occurrence:** recordedBy: John O. Solem; individualCount: 1; sex: female; **Taxon:** order: Hymenoptera; family: Ichneumonidae; genus: Ctenopelma; specificEpithet: lapponicum; scientificNameAuthorship: Holmgren, 1857; **Location:** country: Norway; stateProvince: Oppland; verbatimLocality: Dørålsæter; **Identification:** identifiedBy: Alexey Reshchikov; **Event:** eventDate: 25.VI.1986; **Record Level:** institutionCode: NTNU

#### Distribution

Palaearctic; Sweden, Finland and NW Russia (Karelia).

### Ctenopelma nigrum

Holmgren, 1857

#### Materials

- **Type status:** Other material. **Occurrence:** recordedBy: Remharat; individualCount: 1; sex: female; **Taxon:** order: Hymenoptera; family: Ichneumonidae; genus: Ctenopelma; specificEpithet: nigrum; scientificNameAuthorship: Holmgren, 1857; **Location:** country: Norway; stateProvince: Vestfold; verbatimLocality: Larvik; **Identification:** identifiedBy: Alexey Reshchikov; **Event:** eventDate: 25.VI.1911; **Record Level:** institutionCode: ZMUN

#### Distribution

Palaearctic; Sweden, Finland, Latvia and NW Russia (Karelia).

### Ctenopelma tomentosum

(Desvignes, 1856)

#### Materials

- **Type status:** Other material. **Occurrence:** recordedBy: Ove Sørlibråten; individualCount: 1; sex: female; **Taxon:** order: Hymenoptera; family: Ichneumonidae; genus: Ctenopelma; specificEpithet: tomentosum; scientificNameAuthorship: (Desvignes, 1856); **Location:** country: Norway; stateProvince: Østfold; verbatimLocality: Hvaler, Huser; **Identification:** identifiedBy: Alexey Reshchikov; **Event:** eventDate: 8.VIII.2001; **Record Level:** institutionCode: ZMUN

#### Distribution

Palaearctic; Sweden, Finland, Latvia, Lithuania and NW Russia (Karelia).

### Homaspis subalpina

Schmiedeknecht, 1913

#### Materials

- **Type status:** Other material. **Occurrence:** recordedBy: Ove Sørlibråten; individualCount: 1; sex: female; **Taxon:** order: Hymenoptera; family: Ichneumonidae; genus: Homaspis; specificEpithet: subalpina; scientificNameAuthorship: Schmiedeknecht, 1913; **Location:** country: Norway; stateProvince: Hedmark; verbatimLocality: Kongsvinger, Vennerberg, Langerutberget; **Identification:** identifiedBy: Alexey Reshchikov; **Event:** eventDate: 3.VI.2005; **Record Level:** institutionCode: ZMUN

#### Distribution

Palaearctic; Finland and NW Russia (Karelia).

### Xenoschesis (Polycinetis) fulvicornis

(Kriechbaumer, 1891)

#### Materials

- **Type status:** Other material. **Occurrence:** recordedBy: Ove Sørlibråten; individualCount: 1; sex: female; **Taxon:** order: Hymenoptera; family: Ichneumonidae; genus: Xenochesis; subgenus: Polycinetis; specificEpithet: fulvicornis; scientificNameAuthorship: (Kriechbaumer, 1891); **Location:** country: Norway; stateProvince: Hedmark; verbatimLocality: Eidskog, Vestmarka, Hornåsen; **Identification:** identifiedBy: Alexey Reshchikov; **Event:** eventDate: 13.VI.2004; **Record Level:** institutionCode: ZMUN

#### Distribution

Western Palaearctic; Finland and Latvia.

### Euryproctini

Thomson, 1883

### Euryproctus geniculosus

(Gravenhorst, 1829)

#### Materials

- **Type status:** Other material. **Occurrence:** recordedBy: A. Bakke; individualCount: 1; sex: female; **Taxon:** order: Hymenoptera; family: Ichneumonidae; genus: Euryproctus; specificEpithet: geniculosus; scientificNameAuthorship: (Gravenhorst, 1829); **Location:** country: Norway; stateProvince: Aust-Agder; verbatimLocality: Arendal, Hissey; **Identification:** identifiedBy: Alexey Reshchikov; **Event:** eventDate: 18.VII.1954; **Record Level:** institutionCode: ZMUN

#### Distribution

Western Palaearctic; Denmark, Finland, Sweden, Latvia, Lithuania and NW Russia (Leningrad region).

### Syndipnus pannicularius

(Holmgren, 1857)

#### Materials

- **Type status:** Other material. **Occurrence:** recordedBy: John O. Solem; individualCount: 1; sex: female; **Taxon:** order: Hymenoptera; family: Ichneumonidae; genus: Syndipnus; specificEpithet: pannicularius; scientificNameAuthorship: (Holmgren, 1857); **Location:** country: Norway; stateProvince: Hedmark; verbatimLocality: Storelvdal, Atnaelv, Solbakken; **Identification:** identifiedBy: Alexey Reshchikov; **Event:** samplingProtocol: Malaise trap; eventDate: 24.VI.1986; **Record Level:** institutionCode: NTNU

#### Distribution

Holarctic; Sweden and Finland.

### Mesoleiini

Thomson, 1883

### Saotis albionis

Kasparyan, 2007

#### Materials

- **Type status:** Other material. **Occurrence:** recordedBy: John O. Solem; individualCount: 2; sex: females; **Taxon:** order: Hymenoptera; family: Ichneumonidae; genus: Saotis; specificEpithet: albionis; scientificNameAuthorship: Kasparyan, 2007; **Location:** country: Norway; stateProvince: Hedmark; verbatimLocality: Atnaelv, Solbakken; **Identification:** identifiedBy: Alexey Reshchikov; **Event:** samplingProtocol: Malaise trap; eventDate: 24.VI.1986; **Record Level:** institutionCode: NTNU

#### Distribution

Holarctic; United Kingdom, Finland.

### Saotis heteropus

(Thomson, 1883)

#### Materials

- **Type status:** Other material. **Occurrence:** recordedBy: John O. Solem; individualCount: 1; sex: female; **Taxon:** order: Hymenoptera; family: Ichneumonidae; genus: Saotis; specificEpithet: heteropus; scientificNameAuthorship: (Thomson, 1883); **Location:** country: Norway; stateProvince: Hedmark; verbatimLocality: Atnaelv, Solbakken; **Identification:** identifiedBy: Alexey Reshchikov; **Event:** samplingProtocol: Malaise trap; eventDate: 24.VI.1986; **Record Level:** institutionCode: NTNU

#### Distribution

Holarctic; Sweden, Finland and NW Russia (Leningrad region).

### Saotis pygidiator arctor

Kasparyan, 2009

#### Materials

- **Type status:** Other material. **Occurrence:** recordedBy: H. Elven; individualCount: 1; sex: female; **Taxon:** order: Hymenoptera; family: Ichneumonidae; genus: Saotis; specificEpithet: pygidiator; infraspecificEpithet: arctor; scientificNameAuthorship: Kasparyan, 2009; **Location:** country: Norway; stateProvince: Nordland; verbatimLocality: Bodø, Nordstranda, Skjelstadt; **Identification:** identifiedBy: Alexey Reshchikov; **Event:** eventDate: 27.VI.1995; **Record Level:** institutionCode: UEF

#### Distribution

Western Palaearctic.

#### Notes

New to Scandinavia.

### Perilissini

Thomson, 1883

### Perilissus compressus

Thomson, 1883

#### Materials

- **Type status:** Other material. **Occurrence:** recordedBy: John O. Solem; individualCount: 1; sex: female; **Taxon:** order: Hymenoptera; family: Ichneumonidae; genus: Perilissus; specificEpithet: compressus; scientificNameAuthorship: Thomson, 1883; **Location:** country: Norway; stateProvince: Hedmark; verbatimLocality: Storelvdal, Atnaelv, Solbakken; **Identification:** identifiedBy: Alexey Reshchikov; **Event:** samplingProtocol: Malaise trap; eventDate: 24.VI.1986; **Record Level:** institutionCode: NTNU

#### Distribution

Western Palaearctic; Sweden and Finland.

### Synoecetes anterior

(Thomson, 1894)

#### Materials

- **Type status:** Other material. **Occurrence:** recordedBy: John O. Solem; individualCount: 1; sex: female; **Taxon:** order: Hymenoptera; family: Ichneumonidae; **Location:** country: Norway; stateProvince: Hedmark; verbatimLocality: Storelvdal, Atnaelv, Solbakken; **Identification:** identifiedBy: Alexey Reshchikov; **Event:** samplingProtocol: Malaise trap; eventDate: 24.VI.1986; **Record Level:** institutionCode: NTNU

#### Distribution

Western Palaearctic; Sweden.

### Pionini

Smith & Shenefelt, 1955

### Asthenara socia

(Holmgren, 1857)

#### Materials

- **Type status:** Other material. **Occurrence:** recordedBy: K. Aagaard; individualCount: 8; sex: 7 females, 1 male; **Taxon:** order: Hymenoptera; family: Ichneumonidae; genus: Asthenara; specificEpithet: socia; scientificNameAuthorship: (Holmgren, 1857); **Location:** country: Norway; stateProvince: Nord-Trøndelag; verbatimLocality: Hoylandet, Tverråa stream; verbatimElevation: 340 m; verbatimLatitude: 64.39° N; verbatimLongitude: 12.08° E; **Identification:** identifiedBy: Alexey Reshchikov; **Event:** eventDate: 18.VII.1986; **Record Level:** institutionCode: NTNU

#### Distribution

Western Palaearctic; Sweden, Finland, Lithuania and NW Russia (Karelia).

### Glyptorhaestus punctatus

(Thomson, 1890)

#### Materials

- **Type status:** Other material. **Occurrence:** recordedBy: L.O. Hansen; individualCount: 1; sex: female; **Taxon:** order: Hymenoptera; family: Ichneumonidae; genus: Glyptorhaestus; specificEpithet: punctatus; scientificNameAuthorship: (Thomson, 1890); **Location:** country: Norway; stateProvince: Akerhus; verbatimLocality: Oslo, Ekebergsraninger; **Identification:** identifiedBy: Alexey Reshchikov; **Event:** samplingProtocol: Malaise trap; eventDate: 29.V-27.VI.2007; **Record Level:** institutionCode: ZMUN

#### Distribution

Palaearctic; Sweden, Finland, Lithuania and Latvia.

### Lethades lapponator

Hinz, 1976

#### Materials

- **Type status:** Other material. **Occurrence:** recordedBy: John O. Solem; individualCount: 4; sex: females; **Taxon:** order: Hymenoptera; family: Ichneumonidae; genus: Lethades; specificEpithet: lapponator; scientificNameAuthorship: Hinz, 1976; **Location:** country: Norway; stateProvince: Sør-Trøndelag; verbatimLocality: Oppdal, Kongsvoll, Gavalibk; **Identification:** identifiedBy: Alexey Reshchikov; **Event:** eventDate: 13.VII.1982; **Record Level:** institutionCode: NTNU

#### Distribution

Western Palaerctic; Sweden.

### Rhaestus ophthalmicus

(Holmgren, 1857)

#### Materials

- **Type status:** Other material. **Occurrence:** recordedBy: John O. Solem; individualCount: 4; sex: males; **Taxon:** order: Hymenoptera; family: Ichneumonidae; genus: Rhaestus; specificEpithet: ophthalmicus; scientificNameAuthorship: (Holmgren, 1857); **Location:** country: Norway; stateProvince: Sør-Trøndelag; verbatimLocality: Oppdal, Kongsvoll, Blesbekken; **Identification:** identifiedBy: Alexey Reshchikov; **Event:** samplingProtocol: Malaise trap; eventDate: 10.VII.1980; **Record Level:** institutionCode: NTNU

#### Distribution

Palaearctic; Sweden, Finland and NW Russia (Karelia, Leningrad region).

### Sympherta canaliculata

(Thomson, 1893)

#### Materials

- **Type status:** Other material. **Occurrence:** recordedBy: John O. Solem; individualCount: 1; sex: female; **Taxon:** order: Hymenoptera; family: Ichneumonidae; genus: Sympherta; specificEpithet: canaliculata; scientificNameAuthorship: (Thomson, 1893); **Location:** country: Norway; stateProvince: Hedmark; verbatimLocality: Folldal, Atnaelv, Vollen; **Identification:** identifiedBy: Alexey Reshchikov; **Event:** samplingProtocol: Malaise trap; eventDate: 18-25.VI.1986; **Record Level:** institutionCode: NTNU

#### Distribution

Western Palaearctic; Lithuania.

### Sympherta jactator

(Thunberg, 1824)

#### Materials

- **Type status:** Other material. **Occurrence:** recordedBy: John O. Solem; individualCount: 13; sex: 3 females, 10 males; **Taxon:** order: Hymenoptera; family: Ichneumonidae; genus: Sympherta; specificEpithet: jactator; scientificNameAuthorship: (Thunberg, 1824); **Location:** country: Norway; stateProvince: Hedmark; verbatimLocality: Storelvdal, Solbakken; **Identification:** identifiedBy: Alexey Reshchikov; **Event:** samplingProtocol: Malaise trap; eventDate: 24.VIII.1986; **Record Level:** institutionCode: NTNU

#### Distribution

Western Palaearctic; Sweden and NW Russia (Karelia).

### Sympherta splendens

(Strobl, 1903)

#### Materials

- **Type status:** Other material. **Occurrence:** recordedBy: Ole Lønnve; individualCount: 1; sex: female; **Taxon:** order: Hymenoptera; family: Ichneumonidae; genus: Sympherta; specificEpithet: splendens; scientificNameAuthorship: (Strobl, 1903); **Location:** country: Norway; stateProvince: Akerhus; verbatimLocality: Oppegård, Svartskog, Rødstein; **Identification:** identifiedBy: Alexey Reshchikov; **Event:** samplingProtocol: Malaise trap; eventDate: 4.VI-4.VII.2004; **Record Level:** institutionCode: ZMUN

#### Distribution

Palaearctic; Finland and Lithuania.

### Sympherta sulcata

(Thomson, 1890)

#### Materials

- **Type status:** Other material. **Occurrence:** recordedBy: Ole Lønnve; individualCount: 1; sex: female; **Taxon:** order: Hymenoptera; family: Ichneumonidae; genus: Sympherta; specificEpithet: sulcata; scientificNameAuthorship: (Thomson, 1890); **Location:** country: Norway; stateProvince: Akerhus; verbatimLocality: Oppegård, Svartskog, Rødstein; **Identification:** identifiedBy: Alexey Reshchikov; **Event:** samplingProtocol: Malaise trap; eventDate: 4.VI-4.VII.2004; **Record Level:** institutionCode: ZMUN

#### Distribution

Palaearctic; Finland, Sweden and NW Russia (Karelia).

### Trematopygus rufator

Hinz, 1986

#### Materials

- **Type status:** Other material. **Occurrence:** recordedBy: John O. Solem; individualCount: 1; sex: female; **Taxon:** order: Hymenoptera; family: Ichneumonidae; genus: Trematopygus; specificEpithet: rufator; scientificNameAuthorship: Hinz, 1986; **Location:** country: Norway; stateProvince: Nord-Trøndelag; verbatimLocality: Høylandet, Tverråa stream; **Identification:** identifiedBy: Alexey Reshchikov; **Event:** samplingProtocol: Malaise trap; eventDate: 25.VI.1986; **Record Level:** institutionCode: NTNU

#### Distribution

Western Palaearctic; Sweden and Finland.

### Trematopygus terebrator

Hinz, 1986

#### Materials

- **Type status:** Other material. **Occurrence:** recordedBy: John O. Solem; individualCount: 1; sex: female; **Taxon:** order: Hymenoptera; family: Ichneumonidae; genus: Trematopygus; specificEpithet: terebrator; scientificNameAuthorship: Hinz, 1986; **Location:** country: Norway; stateProvince: Nord-Trøndelag; verbatimLocality: Høylandet, Tverråa stream; **Identification:** identifiedBy: Alexey Reshchikov; **Event:** samplingProtocol: Malaise trap; eventDate: 25.VI.1986; **Record Level:** institutionCode: NTNU

#### Distribution

Western Palaearctic; Sweden and Finland.

### Cylloceriinae

Wahl, 1990

### Cylloceria caligata

(Gravenhorst, 1829)

#### Materials

- **Type status:** Other material. **Occurrence:** recordedBy: John O. Solem; individualCount: 6; sex: males; **Taxon:** order: Hymenoptera; family: Ichneumonidae; genus: Cylloceria; specificEpithet: caligata; scientificNameAuthorship: (Gravenhorst, 1829); **Location:** country: Norway; stateProvince: Hedmark; verbatimLocality: Folldal, Vollen; **Identification:** identifiedBy: Andrei E. Humala; **Event:** samplingProtocol: Malaise trap; eventDate: 8.VII.1986; **Record Level:** institutionCode: NTNU
- **Type status:** Other material. **Occurrence:** recordedBy: John O. Solem; individualCount: 2; sex: males; **Taxon:** order: Hymenoptera; family: Ichneumonidae; genus: Cylloceria; specificEpithet: caligata; scientificNameAuthorship: (Gravenhorst, 1829); **Location:** country: Norway; stateProvince: Hedmark; verbatimLocality: Folldal, Vollen; **Identification:** identifiedBy: Andrei E. Humala; **Event:** samplingProtocol: Malaise trap; eventDate: 23.VIII.1986; **Record Level:** institutionCode: NTNU
- **Type status:** Other material. **Occurrence:** recordedBy: E. Stur; individualCount: 1; sex: male; **Taxon:** order: Hymenoptera; family: Ichneumonidae; genus: Cylloceria; specificEpithet: caligata; scientificNameAuthorship: (Gravenhorst, 1829); **Location:** country: Norway; stateProvince: Hedmark; verbatimLocality: Folldal, Vollen; verbatimElevation: 720 m; verbatimLatitude: 61°58'52" N; verbatimLongitude: 10°01'25" E; **Identification:** identifiedBy: Andrei E. Humala; **Event:** samplingProtocol: Malaise trap; eventDate: 11-18.VIII.2008; **Record Level:** institutionCode: NTNU
- **Type status:** Other material. **Occurrence:** recordedBy: L.O. Hansen and E. Rindal; individualCount: 1; sex: female; **Taxon:** order: Hymenoptera; family: Ichneumonidae; genus: Cylloceria; specificEpithet: caligata; scientificNameAuthorship: (Gravenhorst, 1829); **Location:** country: Norway; stateProvince: Hedmark; verbatimLocality: Elverum, Starmoen NR (N); **Identification:** identifiedBy: Andrei E. Humala; **Event:** samplingProtocol: Malaise trap; eventDate: 29.VII-14.IX.2004; **Record Level:** institutionCode: ZMUN
- **Type status:** Other material. **Occurrence:** recordedBy: E. Heibo and O. Lønnve; individualCount: 1; sex: male; **Taxon:** order: Hymenoptera; family: Ichneumonidae; genus: Cylloceria; specificEpithet: caligata; scientificNameAuthorship: (Gravenhorst, 1829); **Location:** country: Norway; stateProvince: Akershus; verbatimLocality: Hurdal, Brustad; **Identification:** identifiedBy: Andrei E. Humala; **Event:** eventDate: 17.VIII.1996; **Record Level:** institutionCode: ZMUN
- **Type status:** Other material. **Occurrence:** recordedBy: L.O. Hansen; individualCount: 12; sex: males; **Taxon:** order: Hymenoptera; family: Ichneumonidae; genus: Cylloceria; specificEpithet: caligata; scientificNameAuthorship: (Gravenhorst, 1829); **Location:** country: Norway; stateProvince: Akershus; verbatimLocality: Ullensaker, Sessvollmoen, Aurtjernet W.; **Identification:** identifiedBy: Andrei E. Humala; **Event:** samplingProtocol: Malaise trap; eventDate: 26.VI-25.VIII.2008; **Record Level:** institutionCode: ZMUN
- **Type status:** Other material. **Occurrence:** recordedBy: L.O. Hansen; individualCount: 22; sex: females; **Taxon:** order: Hymenoptera; family: Ichneumonidae; genus: Cylloceria; specificEpithet: caligata; scientificNameAuthorship: (Gravenhorst, 1829); **Location:** country: Norway; stateProvince: Akershus; verbatimLocality: Ullensaker, Sessvollmoen, Aurtjernet W.; **Identification:** identifiedBy: Andrei E. Humala; **Event:** samplingProtocol: Malaise trap; eventDate: 26.VI-25.VIII.2008; **Record Level:** institutionCode: ZMUN
- **Type status:** Other material. **Occurrence:** recordedBy: L.O. Hansen; individualCount: 3; sex: females; **Taxon:** order: Hymenoptera; family: Ichneumonidae; genus: Cylloceria; specificEpithet: caligata; scientificNameAuthorship: (Gravenhorst, 1829); **Location:** country: Norway; stateProvince: Akershus; verbatimLocality: Ullensaker, Sessvollmoen, Aurtjernet W.; **Identification:** identifiedBy: Andrei E. Humala; **Event:** samplingProtocol: Malaise trap; eventDate: 30.VIII-1.X.2008; **Record Level:** institutionCode: ZMUN
- **Type status:** Other material. **Occurrence:** recordedBy: L.O. Hansen; individualCount: 7; sex: males; **Taxon:** order: Hymenoptera; family: Ichneumonidae; genus: Cylloceria; specificEpithet: caligata; scientificNameAuthorship: (Gravenhorst, 1829); **Location:** country: Norway; stateProvince: Akershus; verbatimLocality: Ullensaker, Sessvollmoen, Aurtjernet W.; **Identification:** identifiedBy: Andrei E. Humala; **Event:** samplingProtocol: Malaise trap; eventDate: 30.VIII-1.X.2008; **Record Level:** institutionCode: ZMUN
- **Type status:** Other material. **Occurrence:** recordedBy: L.O. Hansen; individualCount: 9; sex: males; **Taxon:** order: Hymenoptera; family: Ichneumonidae; genus: Cylloceria; specificEpithet: caligata; scientificNameAuthorship: (Gravenhorst, 1829); **Location:** country: Norway; stateProvince: Buskerud; verbatimLocality: Nedre-Eiker, Mjøndalen, Ryggkollen (W); **Identification:** identifiedBy: Andrei E. Humala; **Event:** samplingProtocol: Malaise trap; eventDate: 3.VIII-28.IX.2008; **Record Level:** institutionCode: ZMUN
- **Type status:** Other material. **Occurrence:** recordedBy: L.O. Hansen; individualCount: 5; sex: females; **Taxon:** order: Hymenoptera; family: Ichneumonidae; genus: Cylloceria; specificEpithet: caligata; scientificNameAuthorship: (Gravenhorst, 1829); **Location:** country: Norway; stateProvince: Buskerud; verbatimLocality: Nedre-Eiker, Mjøndalen, Ryggkollen (W); **Identification:** identifiedBy: Andrei E. Humala; **Event:** samplingProtocol: Malaise trap; eventDate: 3.VIII-28.IX.2008; **Record Level:** institutionCode: ZMUN

#### Distribution

Holarctic; Finland, Sweden, Lithuania and NW Russia (Karelia).

#### Notes

This species was reported for Norway several times (Ulbricht 1912, Strand 1913, Roman 1942, Riedel and Berg 1997) but it was synonymised under *Cylloceria melancholica* Gravenhorst, 1820 without sufficient reasons (Rossem van 1987). According to Humala (2002) *Cylloceria caligata* Gravenhorst is a distinct species, and it should be included again in the list of Norwegian Ichneumonidae.

### Diplazontinae

Viereck, 1918

### Diplazon sp.1

Klopfstein, 2014 (in press)

#### Materials

- **Type status:** Other material. **Occurrence:** recordedBy: John O. Solem; individualCount: 1; sex: male; **Taxon:** order: Hymenoptera; family: Ichneumonidae; genus: Diplazon; specificEpithet: sp.1; **Location:** country: Norway; stateProvince: Nord-Trøndelag; verbatimLocality: Høylandet, Skiftesåa stream; **Identification:** identifiedBy: Seraina Klopfstein; **Event:** eventDate: 1.VII.1986; **Record Level:** institutionCode: NTNU

#### Distribution

Western Palaearctic; Norway and Sweden.

#### Notes

The description of this new species will be published soon (Klopfstein 2014 in press).

### Homotropus melanogaster

(Holmgren, 1872)

#### Materials

- **Type status:** Other material. **Occurrence:** recordedBy: Strand; individualCount: 1; sex: female; **Taxon:** order: Hymenoptera; family: Ichneumonidae; genus: Homotropus; specificEpithet: melanogaster; scientificNameAuthorship: (Holmgren, 1872); **Location:** country: Norway; stateProvince: Nordland; verbatimLocality: Vefsn, Røsvaud; **Identification:** identifiedBy: Seraina Klopfstein; **Record Level:** institutionCode: ZMUN

#### Distribution

Holarctic; Sweden.

### Sussaba hinzi

Diller, 1982

#### Materials

- **Type status:** Other material. **Occurrence:** recordedBy: K. Sand; individualCount: 1; sex: female; **Taxon:** order: Hymenoptera; family: Ichneumonidae; genus: Sussaba; specificEpithet: hinzi; scientificNameAuthorship: Diller, 1982; **Location:** country: Norway; stateProvince: Oppland; verbatimLocality: Øystre Slidre, Heimdalen; **Identification:** identifiedBy: Seraina Klopfstein; **Event:** eventDate: 2-3.VIII.1995; **Record Level:** institutionCode: ZMUN

#### Distribution

Palaearctic; Sweden, NW Russia (Leningrad region).

### Syrphoctonus idari

Diller, 1985

#### Materials

- **Type status:** Other material. **Occurrence:** recordedBy: John O. Solem; individualCount: 1; sex: female; **Taxon:** order: Hymenoptera; family: Ichneumonidae; genus: Syrphoctonus; specificEpithet: idari; scientificNameAuthorship: Diller, 1985; **Location:** country: Norway; stateProvince: Nord-Trøndelag; verbatimLocality: Høylandet, Tverråa stream; **Identification:** identificationID: Seraina Klopfstein; **Event:** samplingProtocol: Malaise trap; eventDate: 25.VI.1986; **Record Level:** institutionCode: NTNU

#### Distribution

Western Palaearctic; Sweden.

### Syrphophilus asperatus

Dash, 1964

#### Materials

- **Type status:** Other material. **Occurrence:** recordedBy: L.O. Hansen; individualCount: 1; sex: female; **Taxon:** order: Hymenoptera; family: Ichneumonidae; genus: Syrphophilus; specificEpithet: asperatus; scientificNameAuthorship: Dash, 1964; **Location:** country: Norway; stateProvince: Akerhus; verbatimLocality: Ullensaker, Sessvollmoen W; verbatimElevation: 204 m; **Identification:** identifiedBy: Seraina Klopfstein; **Event:** samplingProtocol: Malaise trap; eventDate: 11-26.VI.2007; habitat: sandy pine forest; **Record Level:** institutionCode: ZMUN
- **Type status:** Other material. **Occurrence:** recordedBy: E. Heibo & O. Lønnve; individualCount: 1; sex: female; **Taxon:** order: Hymenoptera; family: Ichneumonidae; genus: Syrphophilus; specificEpithet: asperatus; scientificNameAuthorship: Dash, 1964; **Location:** country: Norway; stateProvince: Nordland; verbatimLocality: Saltdal, Junkerdalen; **Identification:** identifiedBy: Seraina Klopfstein; **Event:** samplingProtocol: Malaise trap; eventDate: 31.V-29.VI.2002; habitat: sandy pine forest; **Record Level:** institutionCode: ZMUN

#### Distribution

Holarctic; Sweden.

### Woldstedtius holarcticus

(Diller, 1969)

#### Materials

- **Type status:** Other material. **Occurrence:** recordedBy: L.O. Hansen & E. Rindal; individualCount: 1; sex: female; **Taxon:** order: Hymenoptera; family: Ichneumonidae; genus: Woldstedtius; specificEpithet: holarcticus; scientificNameAuthorship: (Diller, 1969); **Location:** country: Norway; stateProvince: Hedmark; verbatimLocality: Elverum, Starmoen NR (N); **Identification:** identificationID: Seraina Klopfstein; **Event:** samplingProtocol: Malaise trap; eventDate: 11.VI -29.VII.2004; **Record Level:** institutionCode: ZMUN

#### Distribution

Holarctic and Oriental; Finland.

### Ichneumoninae

Latreille, 1802

### Ichneumonini

Latreille, 1802

### Patrocloides diasemae

(Tischbein, 1877)

#### Materials

- **Type status:** Other material. **Occurrence:** recordedBy: Jon Kr. Skei; individualCount: 2; sex: females; **Taxon:** order: Hymenoptera; family: Ichneumonidae; genus: Patrocloides; specificEpithet: diasemae; scientificNameAuthorship: (Tischbein, 1877); **Location:** country: Norway; stateProvince: Sør-Trøndelag; verbatimLocality: Trondheim: Gjeddvatnet; **Identification:** identifiedBy: Andrei E. Humala; **Event:** samplingProtocol: Malaise trap 4; eventDate: 6.VI.2010; **Record Level:** institutionCode: NTNU
- **Type status:** Other material. **Occurrence:** recordedBy: Jon Kr. Skei; individualCount: 1; sex: female; **Taxon:** order: Hymenoptera; family: Ichneumonidae; genus: Patrocloides; specificEpithet: diasemae; scientificNameAuthorship: (Tischbein, 1877); **Location:** country: Norway; stateProvince: Sør-Trøndelag; verbatimLocality: Kjerringtjønna; **Identification:** identifiedBy: Andrei E. Humala; **Event:** samplingProtocol: Malaise trap 1; eventDate: 6.VI.2010; **Record Level:** institutionCode: NTNU

#### Distribution

Palaearctic; Finland, Sweden and NW Russia (Karelia).

### Metopiinae

Förster, 1869

### Exochus ferus

Tolkanitz, 1993

#### Materials

- **Type status:** Other material. **Occurrence:** recordedBy: Andrei E. Humala & Alexey Reshchikov; individualCount: 1; sex: male; **Taxon:** order: Hymenoptera; family: Ichneumonidae; genus: Exochus; specificEpithet: ferus; scientificNameAuthorship: Tolkanitz, 1993; **Location:** country: Norway; stateProvince: Sør-Trøndelag; verbatimLocality: Åfjord, Skjerva Nature Reserve; verbatimElevation: 42 m; verbatimLatitude: 63°56'36.6" N; verbatimLongitude: 10°16'21.0" E; **Identification:** identifiedBy: Andrei E. Humala; **Event:** samplingProtocol: Malaise trap; eventDate: 9.VII-03.IX.2011; habitat: birch forest; **Record Level:** institutionCode: ZMUN

#### Distribution

Palaearctic.

#### Notes

New to Scandinavia.

### Exochus kuslitzkyi

Tolkanitz, 2003

#### Materials

- **Type status:** Other material. **Occurrence:** recordedBy: Andrei E. Humala & Alexey Reshchikov; individualCount: 1; sex: male; associatedSequences: voucher for DNA barcoding AH074; **Taxon:** order: Hymenoptera; family: Ichneumonidae; genus: Exochus; specificEpithet: kuslitzkyi; scientificNameAuthorship: Tolkanitz, 2003; **Location:** country: Norway; stateProvince: Hordaland; verbatimLocality: Stord, Leirvik, Gullberg; verbatimElevation: 82 m; verbatimLatitude: 59°46'22.4" N; verbatimLongitude: 05°30'42.0" E; **Identification:** identifiedBy: Andrei E. Humala; **Event:** samplingProtocol: Malaise trap; eventDate: 6.VII-28.VIII.2011; habitat: broad-leaved forest; **Record Level:** institutionCode: ZMUN
- **Type status:** Other material. **Occurrence:** recordedBy: Alexey Reshchikov; individualCount: 1; sex: male; **Taxon:** order: Hymenoptera; family: Ichneumonidae; genus: Exochus; specificEpithet: kuslitzkyi; scientificNameAuthorship: Tolkanitz, 2003; **Location:** country: Norway; stateProvince: Vest-Agder; verbatimLocality: Farsund, Listeid Nature Reserve; verbatimElevation: 50-100 m; verbatimLatitude: 58°07'55'' N; verbatimLongitude: 06°42'57'' E; **Identification:** identifiedBy: Andrei E. Humala; **Event:** samplingProtocol: netting; eventDate: 1.VII.2011; habitat: oak forest; **Record Level:** institutionCode: ZMUN
- **Type status:** Other material. **Occurrence:** recordedBy: Alexey Reshchikov; individualCount: 1; sex: male; **Taxon:** order: Hymenoptera; family: Ichneumonidae; genus: Exochus; specificEpithet: kuslitzkyi; scientificNameAuthorship: Tolkanitz, 2003; **Location:** country: Norway; stateProvince: Rogaland; verbatimLocality: Tysvær, Nedstrand, Leirå, Tunganeset; verbatimLatitude: 59°20'36" N; verbatimLongitude: 05°52'35" E; **Identification:** identifiedBy: Andrei E. Humala; **Event:** samplingProtocol: netting; eventDate: 5.VII.2011; habitat: oak forest; **Record Level:** institutionCode: ZMUN

#### Distribution

Palaearctic.

#### Notes

New to Scandinavia.

### Exochus lictor

Haliday, 1839

#### Materials

- **Type status:** Other material. **Occurrence:** recordedBy: Andrei E. Humala & Alexey Reshchikov; individualCount: 1; sex: male; **Taxon:** order: Hymenoptera; family: Ichneumonidae; genus: Exochus; specificEpithet: lictor; scientificNameAuthorship: Haliday, 1839; **Location:** country: Norway; stateProvince: Vest-Agder; verbatimLocality: Farsund, Listeid Nature Reserve; verbatimElevation: 133 m; verbatimLatitude: 58°08'35.2'' N; verbatimLongitude: 06°44'11.3'' E; **Identification:** identifiedBy: Andrei E. Humala; **Event:** samplingProtocol: Malaise trap; eventDate: 1.VII-24.VIII.2011; habitat: oak forest; **Record Level:** institutionCode: ZMUN

#### Distribution

Holarctic; Finland, Latvia, Lithuania and NW Russia (Karelia).

### Exochus lineifrons

Thomson, 1887

#### Materials

- **Type status:** Other material. **Occurrence:** recordedBy: Andrei E. Humala & Alexey Reshchikov; individualCount: 2; sex: females; associatedSequences: voucher for DNA barcoding AH016; **Taxon:** order: Hymenoptera; family: Ichneumonidae; genus: Exochus; specificEpithet: lineifrons; scientificNameAuthorship: Thomson, 1887; **Location:** country: Norway; stateProvince: Rogaland; verbatimLocality: Tysvær, Silgjerd, Åsen; verbatimElevation: 50 m; verbatimLatitude: 59°20'08.6'' N; verbatimLongitude: 05°43'37.6'' E; **Identification:** identifiedBy: Andrei E. Humala; **Event:** samplingProtocol: Malaise trap; eventDate: 5.VII-27.VIII.2011; habitat: oak forest; **Record Level:** institutionCode: ZMUN

#### Distribution

Palaearctic; Finland and Sweden.

### Triclistus globulipes

(Desvignes, 1856)

#### Materials

- **Type status:** Other material. **Occurrence:** recordedBy: Andrei E. Humala & Alexey Reshchikov; individualCount: 1; sex: female; **Taxon:** order: Hymenoptera; family: Ichneumonidae; genus: Triclistus; specificEpithet: globulipes; scientificNameAuthorship: (Desvignes, 1856); **Location:** country: Norway; stateProvince: Aust-Agder; verbatimLocality: Lillesand, Vestre Grimevann lake; verbatimLatitude: 58°19'23.1" N; verbatimLongitude: 08°20'09.1" E; **Identification:** identifiedBy: Andrei E. Humala; **Event:** samplingProtocol: Malaise trap; eventDate: 29.VI-23.VIII.2011; habitat: oak forest; **Record Level:** institutionCode: ZMUN

#### Distribution

Palaearctic and Oriental; Denmark, Finland, Latvia, Lithuania, Sweden and NW Russia (Karelia).

### Pimplinae

Wesmael, 1845

### Delomeristini

Hellén, 1915

### Delomerista longicauda

Kasparyan, 1973

#### Materials

- **Type status:** Other material. **Occurrence:** recordedBy: Kaare Aagaard; individualCount: 1; sex: female; **Taxon:** order: Hymenoptera; family: Ichneumonidae; genus: Delomerista; specificEpithet: longicauda; scientificNameAuthorship: Kasparyan, 1973; **Location:** country: Norway; stateProvince: Nord-Trøndelag; verbatimLocality: Høylandet, Tverråa; verbatimElevation: 340 m; **Identification:** identifiedBy: Andrei E. Humala; **Event:** samplingProtocol: Malaise trap; eventDate: 30.VII.1986; **Record Level:** institutionCode: NTNU

#### Distribution

Palaearctic; Sweden.

### Ephialtini

Hellén, 1915

### Schizopyga pictifrons

(Thomson, 1877)

#### Materials

- **Type status:** Other material. **Occurrence:** recordedBy: Andrei E. Humala & Alexey Reshchikov; individualCount: 1; sex: male; **Taxon:** order: Hymenoptera; family: Ichneumonidae; genus: Schizopyga; specificEpithet: pictifrons; scientificNameAuthorship: (Thomson, 1877); **Location:** country: Norway; stateProvince: Sør-Trøndelag; verbatimLocality: Åfjord, Skjerva Nature Reserve; verbatimElevation: 42 m; verbatimLatitude: 63°56'36.6" N; verbatimLongitude: 10°16'21.0" E; **Identification:** identifiedBy: Andrei E. Humala; **Event:** samplingProtocol: Malaise trap; eventDate: 09.VII-03.IX.2011; habitat: birch forest; **Record Level:** institutionCode: ZMUN
- **Type status:** Other material. **Occurrence:** recordedBy: Alexey Reshchikov; individualCount: 1; sex: male; **Taxon:** order: Hymenoptera; family: Ichneumonidae; genus: Schizopyga; specificEpithet: pictifrons; scientificNameAuthorship: (Thomson, 1877); **Location:** country: Norway; stateProvince: Vest-Agder; verbatimLocality: Farsund, Listeid Nature Reserve; verbatimElevation: 50-150 m; verbatimLatitude: 58°07'55'' N; verbatimLongitude: 06°42'57'' E; **Identification:** identifiedBy: Andrei E. Humala; **Event:** samplingProtocol: netting; eventDate: 01.VII.2011; habitat: oak forest; **Record Level:** institutionCode: ZMUN

#### Distribution

Palaearctic; Finland and Sweden.

### Pimplini

Wesmael, 1845

### Itoplectis insignis

Perkins, 1957

#### Materials

- **Type status:** Other material. **Occurrence:** recordedBy: A. Humala & A. Reshchikov; individualCount: 1; sex: female; **Taxon:** order: Hymenoptera; family: Ichneumonidae; genus: Itoplectis; specificEpithet: insignis; scientificNameAuthorship: Perkins, 1957; **Location:** country: Norway; stateProvince: Sogn og Fjordane; verbatimLocality: Leikanger, Skopargilet, Bargarden Nature Reserve; verbatimElevation: 114 m; verbatimLatitude: 61°12'18.7" N; verbatimLongitude: 06°40'30.4" E; **Identification:** identifiedBy: Alexey Reshchikov; **Event:** samplingProtocol: Malaise trap; eventDate: 7.VII-30.VIII.2011; habitat: broad-leaved forest; **Record Level:** institutionCode: ZMUN

#### Distribution

Palaearctic; Finland and Sweden.

### Poemeniinae

Narayanan & Lal, 1953

### Neoxorides montanus

Oehlke, 1966

#### Materials

- **Type status:** Other material. **Occurrence:** recordedBy: Kaare Aagaard; individualCount: 1; sex: female; **Taxon:** order: Hymenoptera; family: Ichneumonidae; genus: Neoxorides; specificEpithet: montanus; scientificNameAuthorship: Oehlke, 1966; **Location:** country: Norway; stateProvince: Nord-Trøndelag; verbatimLocality: Høylandet, Skiftesåa; Bred inn; verbatimElevation: 200 m; **Identification:** identifiedBy: Andrei E. Humala; **Event:** samplingProtocol: Malaise trap; eventDate: 25.VI.1986; **Record Level:** institutionCode: NTNU
- **Type status:** Other material. **Occurrence:** recordedBy: Kaare Aagaard; individualCount: 1; sex: female; **Taxon:** order: Hymenoptera; family: Ichneumonidae; genus: Neoxorides; specificEpithet: montanus; scientificNameAuthorship: Oehlke, 1966; **Location:** country: Norway; stateProvince: Nord-Trøndelag; verbatimLocality: Høylandet, Skiftesåa; Bred inn; verbatimElevation: 200 m; **Identification:** identifiedBy: Andrei E. Humala; **Event:** samplingProtocol: Malaise trap; eventDate: 15.VII.1987; **Record Level:** institutionCode: NTNU

#### Distribution

Palaearctic; Finland, NW Russia (Leningrad and Murmansk regions).

### Podoschistus scutellaris

(Desvignes, 1856)

#### Materials

- **Type status:** Other material. **Occurrence:** recordedBy: A. Humala & A. Reshchikov; individualCount: 1; sex: female; associatedSequences: voucher for DNA barcoding AVR064; **Taxon:** order: Hymenoptera; family: Ichneumonidae; genus: Podoschistus; specificEpithet: scutellaris; scientificNameAuthorship: (Desvignes, 1856); **Location:** country: Norway; stateProvince: Aust-Agder; verbatimLocality: Lillesand, Vestre Grimevann; verbatimElevation: 78 m; verbatimLatitude: 58°19'23.1" N; verbatimLongitude: 08°20'9.1" E; **Identification:** identifiedBy: Alexey Reshchikov; **Event:** samplingProtocol: Malaise trap; eventDate: 29.VI-23.VIII.2011; habitat: oak forest; **Record Level:** institutionCode: ZMUN

#### Distribution

Palaearctic and Oriental; Sweden and NW Russia.

### Tersilochinae

Schmiedeknecht, 1910

### Diaparsis (Nanodiaparsis) aperta

(Thomson, 1889)

#### Materials

- **Type status:** Other material. **Occurrence:** recordedBy: A. Endrestøl and S. Olberg; individualCount: 2; sex: females; **Taxon:** order: Hymenoptera; family: Ichneumonidae; genus: Diaparsis; subgenus: Nanodiaparsis; specificEpithet: aperta; scientificNameAuthorship: (Thomson, 1889); **Location:** country: Norway; stateProvince: Akerhus; verbatimLocality: Oslo, Bygdøy, Rodeløkken; **Identification:** identifiedBy: Andrey Khalaim; **Event:** samplingProtocol: Malaise trap; eventDate: 27.VI-25.VII.2006; **Record Level:** institutionCode: ZMUN

#### Distribution

Palaearctic; Sweden and Finland.

### Diaparsis (Diaparsis) carinifer

(Thomson, 1889)

#### Materials

- **Type status:** Other material. **Occurrence:** recordedBy: A. Endrestøl; individualCount: 1; sex: female; **Taxon:** order: Hymenoptera; family: Ichneumonidae; genus: Diaparsis; subgenus: Diaparsis; specificEpithet: carinifer; scientificNameAuthorship: (Thomson, 1889); **Location:** country: Norway; stateProvince: Akerhus; verbatimLocality: Oslo, Lindøya; **Identification:** identifiedBy: Andrey Khalaim; **Event:** samplingProtocol: Malaise trap; eventDate: 19.VI-20.VII.2006; **Record Level:** institutionCode: ZMUN

#### Distribution

Palaearctic; Sweden, Finland and Lithuania.

### Diaparsis (Diaparsis) jucunda

(Holmgren, 1860)

#### Materials

- **Type status:** Other material. **Occurrence:** recordedBy: Andrei E. Humala & Alexey Reshchikov; individualCount: 2; sex: females; **Taxon:** order: Hymenoptera; family: Ichneumonidae; genus: Diaparsis; subgenus: Diaparsis; specificEpithet: jucunda; scientificNameAuthorship: (Holmgren, 1860); **Location:** country: Norway; stateProvince: Sogn og Fjordane; verbatimLocality: Leikanger, Kvinnefossen waterfall; verbatimElevation: 60 m; verbatimLatitude: 61°12'30.2" N; verbatimLongitude: 06°38'52.0" E; **Identification:** identifiedBy: Andrey Khalaim; **Event:** samplingProtocol: Malaise trap; eventDate: 6.VII-30.VIII.2011; habitat: spruce forest with juniper; **Record Level:** institutionCode: ZMUN
- **Type status:** Other material. **Occurrence:** recordedBy: Andrei E. Humala & Alexey Reshchikov; individualCount: 2; sex: females; associatedSequences: voucher for DNA barcoding AH060; **Taxon:** order: Hymenoptera; family: Ichneumonidae; genus: Diaparsis; subgenus: Diaparsis; specificEpithet: jucunda; scientificNameAuthorship: (Holmgren, 1860); **Location:** country: Norway; stateProvince: Sogn og Fjordane; verbatimLocality: Leikanger, Skopargilet, Bargarden Nature Reserve; verbatimElevation: 114 m; verbatimLatitude: 61°12'18.7" N; verbatimLongitude: 06°40'30.4" E; **Identification:** identifiedBy: Andrey Khalaim; **Event:** samplingProtocol: Malaise trap; eventDate: 7.VII-30.VIII.2011; habitat: broad-leaved forest; **Record Level:** institutionCode: ZMUN

#### Distribution

Palaearctic; Denmark, Finland, Sweden, and NW Russia (Karelia, Leningrad region).

### Epistathmus crassicornis

Horstmann, 1971

#### Materials

- **Type status:** Other material. **Occurrence:** recordedBy: S.A. Jenseg; individualCount: 3; sex: 2 females, 1 male; **Taxon:** order: Hymenoptera; family: Ichneumonidae; genus: Epistathmus; specificEpithet: crassicornis; scientificNameAuthorship: Horstmann, 1971; **Location:** country: Norway; stateProvince: Akerhus; verbatimLocality: Follo, Ås, Rustadmåsan; verbatimLatitude: 59°39'48.4" N; verbatimLongitude: 10°49'15.9" E; **Identification:** identifiedBy: A. Khalaim; **Event:** samplingProtocol: Malaise trap; eventDate: 15.VII-16.VIII.2011; **Record Level:** institutionCode: ZMUN
- **Type status:** Other material. **Occurrence:** recordedBy: Alexey Reshchikov; individualCount: 1; sex: male; **Taxon:** order: Hymenoptera; family: Ichneumonidae; genus: Epistathmus; specificEpithet: crassicornis; scientificNameAuthorship: Horstmann, 1971; **Location:** country: Norway; stateProvince: Hordaland; verbatimLocality: Os, Lyse kloster; verbatimLatitude: 60°13'50.8" N; verbatimLongitude: 05°24'6.9" E; **Identification:** identifiedBy: A. Khalaim; **Event:** samplingProtocol: sweeping net; eventDate: 29.VIII.2011; **Record Level:** institutionCode: ZMUN
- **Type status:** Other material. **Occurrence:** recordedBy: A. Humala & A. Reshchikov; individualCount: 1; sex: female; **Taxon:** order: Hymenoptera; family: Ichneumonidae; genus: Epistathmus; specificEpithet: crassicornis; scientificNameAuthorship: Horstmann, 1971; **Location:** country: Norway; stateProvince: Sogn og Fjordane; verbatimLocality: Leikanger, Kvinnefossen waterfall; verbatimElevation: 60 m; verbatimLatitude: 61°12'30.2" N; verbatimLongitude: 6°38'52.0" E; **Identification:** identifiedBy: A.Khalaim; **Event:** samplingProtocol: Malaise trap; eventDate: 6.VII-30.VIII.2011; habitat: spruce forest with juniper; **Record Level:** institutionCode: ZMUN
- **Type status:** Other material. **Occurrence:** recordedBy: A. Humala & A. Reshchikov; individualCount: 1; sex: male; **Taxon:** order: Hymenoptera; family: Ichneumonidae; genus: Epistathmus; specificEpithet: crassicornis; scientificNameAuthorship: Horstmann, 1971; **Location:** country: Norway; stateProvince: Sogn og Fjordane; verbatimLocality: Leikanger, Skopargilet; verbatimElevation: 114 m; verbatimLatitude: 61°12'18.7" N; verbatimLongitude: 06°40'30.4" E; **Identification:** identifiedBy: A.Khalaim; **Event:** samplingProtocol: Malaise trap; eventDate: 6.VII-30.VIII.2011; habitat: broad-leaved forest; **Record Level:** institutionCode: ZMUN
- **Type status:** Other material. **Occurrence:** recordedBy: A. Humala & A. Reshchikov; individualCount: 3; sex: males; **Taxon:** order: Hymenoptera; family: Ichneumonidae; genus: Epistathmus; specificEpithet: crassicornis; scientificNameAuthorship: Horstmann, 1971; **Location:** country: Norway; stateProvince: Vest-Agder; verbatimLocality: Farsund, Listeid Nature Reserve; verbatimElevation: 72 m; verbatimLatitude: 58°08'01.6" N; verbatimLongitude: 06°43'08.2" E; **Identification:** identifiedBy: A.Khalaim; **Event:** samplingProtocol: Malaise trap; eventDate: 1.VII-24.VIII.2011; habitat: oak forest; **Record Level:** institutionCode: ZMUN
- **Type status:** Other material. **Occurrence:** recordedBy: A. Humala & A. Reshchikov; individualCount: 2; sex: 1 female, 1 male; **Taxon:** order: Hymenoptera; family: Ichneumonidae; **Location:** country: Norway; stateProvince: Vest-Agder; verbatimLocality: Farsund, Listeid Nature Reserve; verbatimElevation: 133 m; verbatimLatitude: 58°08'35.2'' N; verbatimLongitude: 06°44'11.3'' E; **Identification:** identifiedBy: A.Khalaim; **Event:** samplingProtocol: Malaise trap; eventDate: 1.VII-24.VIII.2011; habitat: oak forest; **Record Level:** institutionCode: ZMUN
- **Type status:** Other material. **Occurrence:** recordedBy: A. Humala & A. Reshchikov; individualCount: 4; sex: 2 females, 2 males; **Taxon:** order: Hymenoptera; family: Ichneumonidae; **Location:** country: Norway; stateProvince: Rogaland; verbatimLocality: Tysvær, Silgjerd, Åsen; verbatimElevation: 50 m; verbatimLatitude: 59°20'08.6'' N; verbatimLongitude: 05°43'37.6'' E; **Identification:** identifiedBy: A.Khalaim; **Event:** samplingProtocol: Malaise trap; eventDate: 5.VII-27.VIII.2011; habitat: oak forest; **Record Level:** institutionCode: ZMUN
- **Type status:** Other material. **Occurrence:** recordedBy: A. Humala & A. Reshchikov; individualCount: 2; sex: 1 female, 1 male; **Taxon:** order: Hymenoptera; family: Ichneumonidae; **Location:** country: Norway; stateProvince: Hordaland; verbatimLocality: Stord, Leirvik, Gullberg; verbatimElevation: 82 m; verbatimLatitude: 59°46'22.4" N; verbatimLongitude: 05°30' 42.0" E; **Identification:** identifiedBy: A.Khalaim; **Event:** samplingProtocol: Malaise trap; eventDate: 6.VII-28.VIII.2011; habitat: broad-leaved forest; **Record Level:** institutionCode: ZMUN
- **Type status:** Other material. **Occurrence:** recordedBy: A. Humala & A. Reshchikov; individualCount: 2; sex: 1 female, 1 male; **Taxon:** order: Hymenoptera; family: Ichneumonidae; **Location:** country: Norway; stateProvince: Aust-Agder; verbatimLocality: Lillesand, Vestre Grimevann lake; verbatimLatitude: 58°19'23.1" N; verbatimLongitude: 08°20'09.1" E; **Identification:** identifiedBy: A.Khalaim; **Event:** samplingProtocol: Malaise trap; eventDate: 29.VI-23.VIII.2011; habitat: oak forest; **Record Level:** institutionCode: ZMUN
- **Type status:** Other material. **Occurrence:** recordedBy: A. Humala & A. Reshchikov; individualCount: 1; sex: male; **Taxon:** order: Hymenoptera; family: Ichneumonidae; **Location:** country: Norway; stateProvince: Sør-Trøndelag; verbatimLocality: Åfjord, Skjerva Nature Reserve; verbatimElevation: 42 m; verbatimLatitude: 63°56'36.6'' N; verbatimLongitude: 10°16'21.0" E; **Identification:** identifiedBy: A.Khalaim; **Event:** samplingProtocol: Malaise trap; eventDate: 9.VII-3.IX.2011; habitat: birch forest; **Record Level:** institutionCode: ZMUN

#### Distribution

Palaearctic; Denmark, Finland and NW Russia (Karelia).

### Gelanes simillimus

Horstmann, 1981

#### Materials

- **Type status:** Other material. **Occurrence:** recordedBy: S.A. Jenseg; individualCount: 2; sex: 1 female, 1 male; **Taxon:** order: Hymenoptera; family: Ichneumonidae; genus: Gelanes; specificEpithet: simillimus; scientificNameAuthorship: Horstmann, 1981; **Location:** country: Norway; stateProvince: Akerhus; verbatimLocality: Follo, Ås, Åsmåsan; verbatimElevation: 126 m; verbatimLatitude: 59°40'16.7" N; verbatimLongitude: 10°46'53.9" E; **Identification:** identifiedBy: A.Khalaim; **Event:** samplingProtocol: Malaise trap; eventDate: 16-19.V.2011; **Record Level:** institutionCode: ZMUN
- **Type status:** Other material. **Occurrence:** recordedBy: S.A. Jenseg; individualCount: 2; sex: 1 female, 1 male; **Taxon:** order: Hymenoptera; family: Ichneumonidae; genus: Gelanes; specificEpithet: simillimus; scientificNameAuthorship: Horstmann, 1981; **Location:** country: Norway; stateProvince: Akerhus; verbatimLocality: Follo, Ås, Rustadmåsan; verbatimLatitude: 59°39'48.4" N; verbatimLongitude: 10°49'15.9" E; **Identification:** identifiedBy: A.Khalaim; **Event:** samplingProtocol: Malaise trap; eventDate: 15.VII-16.VIII.2011; **Record Level:** institutionCode: ZMUN

#### Distribution

Palaearctic; Sweden, Finland, Lithuania and NW Russia.

### Probles (Euporizon) extensor

(Aubert, 1971)

#### Materials

- **Type status:** Other material. **Occurrence:** recordedBy: K. Sand; individualCount: 1; sex: female; **Taxon:** order: Hymenoptera; family: Ichneumonidae; genus: Probles; subgenus: Euporizon; specificEpithet: extensor; scientificNameAuthorship: (Aubert, 1971); **Location:** country: Norway; stateProvince: Oppland; verbatimLocality: Slidre, Heimdalen; **Identification:** identifiedBy: A.Khalaim; **Event:** samplingProtocol: Malaise trap; eventDate: 2-3.VIII.1995; **Record Level:** institutionCode: ZMUN

#### Distribution

Western Palaearctic.

#### Notes

New to Scandinavia.

### Tersilochus (Tersilochus) longicornis

(Thomson, 1889)

#### Materials

- **Type status:** Other material. **Occurrence:** recordedBy: Svein Svendsen; individualCount: 3; sex: females; **Taxon:** order: Hymenoptera; family: Ichneumonidae; genus: Tersilochus; subgenus: Tersilochus; specificEpithet: longicornis; scientificNameAuthorship: (Thomson, 1889); **Location:** country: Norway; stateProvince: Aust-Agder; verbatimLocality: Birkenes, Birkeland; **Identification:** identifiedBy: Andrey Khalaim; **Event:** samplingProtocol: Malaise trap; eventDate: V.2006; **Record Level:** institutionCode: ZMUN

#### Distribution

Palaearctic; Sweden, Finland and NW Russia (Leningrad region).

### Tersilochus (Tersilochus) triangularis

Gravenhorst, 1807)

#### Materials

- **Type status:** Other material. **Occurrence:** recordedBy: A. Endrestøl and L.O. Hansen; individualCount: 1; sex: female; **Taxon:** order: Hymenoptera; family: Ichneumonidae; genus: Tersilochus; subgenus: Tersilochus; specificEpithet: triangularis; scientificNameAuthorship: (Gravenhorst, 1807); **Location:** country: Norway; stateProvince: Akerhus; verbatimLocality: Oslo, Ekebergskråningen [NW]; **Identification:** identifiedBy: Andrey Khalaim; **Event:** samplingProtocol: Malaise trap; eventDate: 24.IV-29.V.2007; **Record Level:** institutionCode: ZMUN

#### Distribution

Palaearctic; Sweden, Finland and Lithuania.

### Tryphoninae

Shuckard, 1840

### Exenterini

Förster, 1869

### Eridolius clauseni

(Kerrich, 1962)

#### Materials

- **Type status:** Other material. **Occurrence:** recordedBy: L.O. Hansen; individualCount: 1; sex: female; **Taxon:** order: Hymenoptera; family: Ichneumonidae; genus: Eridolius; specificEpithet: clauseni; scientificNameAuthorship: (Kerrich, 1962); **Location:** country: Norway; stateProvince: Akerhus; verbatimLocality: Baerum, Oksenøya, Oksenøyveinen no.71; **Identification:** identifiedBy: Alexey Reshchikov; **Event:** samplingProtocol: Malaise trap; eventDate: 1.VII-15.VIII.2003; **Record Level:** institutionCode: ZMUN

#### Distribution

Palaearctic and Oriental.

#### Notes

New to Scandinavia. In Western Palaearctic reported from Germany only.

### Eridolius deletus

(Thomson, 1883)

#### Materials

- **Type status:** Other material. **Occurrence:** recordedBy: Garli; individualCount: 1; sex: female; **Taxon:** order: Hymenoptera; family: Ichneumonidae; genus: Eridolius; specificEpithet: deletus; scientificNameAuthorship: (Thomson, 1883); **Location:** country: Norway; stateProvince: Oppland; verbatimLocality: Sør-Aurdal, Kjenstrud; **Identification:** identifiedBy: Alexey Reshchikov; **Event:** eventDate: VII.1995; **Record Level:** institutionCode: ZMUN

#### Distribution

Palaearctic; Sweden, Finland and NW Russia (Karelia).

### Exenterus confusus

Kerrich, 1952

#### Materials

- **Type status:** Other material. **Occurrence:** recordedBy: Andrei E. Humala & Alexey Reshchikov; individualCount: 1; sex: female; **Taxon:** order: Hymenoptera; family: Ichneumonidae; genus: Exenterus; specificEpithet: confusus; scientificNameAuthorship: Kerrich, 1952; **Location:** country: Norway; stateProvince: Sør-Trøndelag; verbatimLocality: Åfjord, Skjerva Nature Reserve; verbatimElevation: 42 m; verbatimLatitude: 63°56'36.6" N; verbatimLongitude: 10°16'21.0" E; **Identification:** identifiedBy: Andrei E. Humala; **Event:** samplingProtocol: Malaise trap; eventDate: 9.VII-3.IX.2011; habitat: birch forest; **Record Level:** institutionCode: ZMUN

#### Distribution

Holarctic; Finland, Sweden and NW Russia (Karelia).

### Tryphonini

Shuckard, 1840

### Polyblastus (Polyblastus) cothurnatus

(Gravenhorst, 1829)

#### Materials

- **Type status:** Other material. **Occurrence:** recordedBy: Andrei E. Humala & Alexey Reshchikov; individualCount: 1; sex: female; **Taxon:** order: Hymenoptera; family: Ichneumonidae; genus: Polyblastus; subgenus: Polyblastus; specificEpithet: cothurnatus; scientificNameAuthorship: (Gravenhorst, 1829); **Location:** country: Norway; stateProvince: Aust-Agder; verbatimLocality: Lillesand, Vestre Grimevann lake; verbatimLatitude: 58°19'23.1" N; verbatimLongitude: 08°20'09.1" E; **Identification:** identifiedBy: Andrei E. Humala; **Event:** samplingProtocol: Malaise trap; eventDate: 29.VI-23.VIII.2011; habitat: oak forest; **Record Level:** institutionCode: ZMUN

#### Distribution

Palaearctic; Denmark, Finland, Sweden, Latvia, Lithuania and NW Russia (Karelia).

## Supplementary Material

XML Treatment for Acaenitinae

XML Treatment for Coleocentrini

XML Treatment for Coleocentrus caligatus

XML Treatment for Adelognathinae

XML Treatment for Adelognathus difformis

XML Treatment for Adelognathus rufithorax

XML Treatment for Adelognathus stelfoxi

XML Treatment for Banchinae

XML Treatment for Glyptini

XML Treatment for Glypta (Glypta) consimilis

XML Treatment for Campopleginae

XML Treatment for Leptocampoplex cremastoides

XML Treatment for Rhimphoctona (Xylophylax) rufocoxalis

XML Treatment for Cryptinae

XML Treatment for Cryptini

XML Treatment for Ateleute linearis

XML Treatment for Ctenopelmatinae

XML Treatment for Ctenopelmatini

XML Treatment for Ctenopelma boreale

XML Treatment for Ctenopelma lapponicum

XML Treatment for Ctenopelma nigrum

XML Treatment for Ctenopelma tomentosum

XML Treatment for Homaspis subalpina

XML Treatment for Xenoschesis (Polycinetis) fulvicornis

XML Treatment for Euryproctini

XML Treatment for Euryproctus geniculosus

XML Treatment for Syndipnus pannicularius

XML Treatment for Mesoleiini

XML Treatment for Saotis albionis

XML Treatment for Saotis heteropus

XML Treatment for Saotis pygidiator arctor

XML Treatment for Perilissini

XML Treatment for Perilissus compressus

XML Treatment for Synoecetes anterior

XML Treatment for Pionini

XML Treatment for Asthenara socia

XML Treatment for Glyptorhaestus punctatus

XML Treatment for Lethades lapponator

XML Treatment for Rhaestus ophthalmicus

XML Treatment for Sympherta canaliculata

XML Treatment for Sympherta jactator

XML Treatment for Sympherta splendens

XML Treatment for Sympherta sulcata

XML Treatment for Trematopygus rufator

XML Treatment for Trematopygus terebrator

XML Treatment for Cylloceriinae

XML Treatment for Cylloceria caligata

XML Treatment for Diplazontinae

XML Treatment for Diplazon sp.1

XML Treatment for Homotropus melanogaster

XML Treatment for Sussaba hinzi

XML Treatment for Syrphoctonus idari

XML Treatment for Syrphophilus asperatus

XML Treatment for Woldstedtius holarcticus

XML Treatment for Ichneumoninae

XML Treatment for Ichneumonini

XML Treatment for Patrocloides diasemae

XML Treatment for Metopiinae

XML Treatment for Exochus ferus

XML Treatment for Exochus kuslitzkyi

XML Treatment for Exochus lictor

XML Treatment for Exochus lineifrons

XML Treatment for Triclistus globulipes

XML Treatment for Pimplinae

XML Treatment for Delomeristini

XML Treatment for Delomerista longicauda

XML Treatment for Ephialtini

XML Treatment for Schizopyga pictifrons

XML Treatment for Pimplini

XML Treatment for Itoplectis insignis

XML Treatment for Poemeniinae

XML Treatment for Neoxorides montanus

XML Treatment for Podoschistus scutellaris

XML Treatment for Tersilochinae

XML Treatment for Diaparsis (Nanodiaparsis) aperta

XML Treatment for Diaparsis (Diaparsis) carinifer

XML Treatment for Diaparsis (Diaparsis) jucunda

XML Treatment for Epistathmus crassicornis

XML Treatment for Gelanes simillimus

XML Treatment for Probles (Euporizon) extensor

XML Treatment for Tersilochus (Tersilochus) longicornis

XML Treatment for Tersilochus (Tersilochus) triangularis

XML Treatment for Tryphoninae

XML Treatment for Exenterini

XML Treatment for Eridolius clauseni

XML Treatment for Eridolius deletus

XML Treatment for Exenterus confusus

XML Treatment for Tryphonini

XML Treatment for Polyblastus (Polyblastus) cothurnatus

## References

[B455008] Achterberg van C., Zwakhals K. (2004). Fauna Europaea: Ichneumonoidea. http://www.faunaeur.org/.

[B454999] Broad G. (2011). Checklist of British and Irish Ichneumonidae (Hymenoptera). http://www.nhm.ac.uk/resources-rx/files/ichneumonidae-checklist-for-web-27752.pdf.

[B451379] Hansen L. O., Humala A., Reschikov A. V. (2010). Ichneumonidae (Hymenoptera) new for the fauna of Norway, Part 4. Norwegian Journal of Entomology.

[B561842] Horstmann K. (2004). Bemerkungen zur Systematik einiger Gattungen der Campopleginae IV (Hymenoptera, Ichneumonidae). Zeitschrift der Arbeitsgemeinschaft Österreichischer Entomologen.

[B451389] Humala A. E. (2002). A review of the Parasitic Wasps of the Genera *Cylloceria* Schiødte, 1838 and *Allomacrus* Förster, 1868 (Hymenoptera, Ichneumonidae) of the Fauna of Russia. Entomologicheskoe Obozrenie.

[B451399] Humala A. E., Reshchikov A. V. (2012). Ichneumonidae (Hymenoptera) new to the Norwegian fauna. Norwegian Journal of Entomology.

[B451409] Kasparyan D. R., Khalaim A. I., Lelej A. S. (2007). Key to the insects of Russia Far East. Volume IV. Neuropteroidea, Mecoptera, Hymenoptera.

[B463563] Kasparyan D. R., Kopelke J. P. (2009). Taxonomic review and key to European ichneumon-flies (Hymenoptera, Ichneumonidae), parasitoids of gall-forming sawflies of the genera *Pontania* Costa, *Phyllocolpa* Benson, and *Euura* Newman (Hymenoptera, Tenthredinidae) on willows: Part I. Entomological Review.

[B459577] Kasparyan D. R., Kopelke J. P. (2010). A taxonomic review of ichneumon-flies (Hymenoptera, Ichneumonidae), parasitoids of gall-forming sawflies (Hymenoptera, Tenthredinidae) on *Salix*. Part II. Review of the Palaearctic species of the genus *Saotis* Förster with description of four new species. Entomological Review.

[B467380] Klopfstein S. (2014). Revision of the Western Palaearctic Diplazontinae (Hymenoptera, Ichneumonidae). European Journal of Taxonomy.

[B561852] Kopelke J. P. (1994). Der Schmarotzerkomplex (Brutparasiten und Parasitoide) der gallenbildenden Pontania-Arten (Insecta: Hymenoptera: Tenthredinidae). Senckenbergiana Biologica.

[B454920] Koponen M., Jussila R., Vikberg V. (1995). A check list of Finnish Hymenoptera, Parasitica, part 1. family Ichneumonidae, subfamilies Pimplinae, Poemeninae, Rhyssinae and Diacritinae. Sahlbergia.

[B459587] Reshchikov Alexey (2013). Two new species of *Lathrolestes* (Hymenoptera, Ichneumonidae) from Norway, northern Russia and Finland with a key to western Palaearctic species. Zootaxa.

[B454930] Riedel M., Berg Ø. (1997). Faunistic Distribution on Norwegian Ichneumonidae (Hymenoptera). Fauna norvegica.

[B454940] Riedel M., Hansen L. O. (2007). Ichneumonidae (Hymenoptera) new for the fauna of Norway. Part 3.. Norwegian Journal of Entomology.

[B454970] Riedel M., Hansen L. O. (2012). Ichneumonidae (Hymenoptera) new for the fauna of Norway. Part 5.. Norwegian Journal of Entomology.

[B487540] Riedel M., Hansen L. O. (2013). Ichneumonidae (Hymenoptera) new for the fauna of Norway. Part 6. Norwegian Journal of Entomology.

[B454950] Riedel M., Hansen L. O., Berg Ø. (2000). Ichneumonidae (Hymenoptera) new for the fauna of Norway. Norwegian Journal of Entomology.

[B454960] Riedel M., Hansen L. O., Berg Ø. (2005). Ichneumonidae (Hymenoptera) new for the fauna of Norway. Part 2. Norwegian Journal of Entomology.

[B455449] Roman A. (1942). List of new finds of Ichneumonidae in Norway. Tromsø Mus. Årsheft. Nat. Avd..

[B454980] Rossem van G. (1987). A Revision of Western Palaearctic Oxytorine Genera. Part VI.. Tijdschrift voor Entomologie.

[B455459] Strand E. (1913). Hymenoptera Parasitica. Neue Beiträge zur Arthropoda-Fauna Norwegens nebst gelegenlichen Bemerkungen über die deutchen Arten. Nyt Mag. Naturvidensk..

[B455469] Ulbricht A. (1912). Pimplinae. In Strand, E., Hymenoptera Parasitica. Neue Beiträge zur Arthropoda-Fauna Norwegens nebst gelegenlichen Bemerkungen über die deutchen Arten. VIII. Nyt Mag. Naturvidensk.

[B454990] Yu D. S., van Achterberg K., Horstmann K. (2012). Taxapad 2012, Ichneumonoidea 2011. Database on flash-drive. Ottawa, Ontario, Canada.. www.taxapad.com.

